# The perspectives of health professionals and patients on racism in healthcare: A qualitative systematic review

**DOI:** 10.1371/journal.pone.0255936

**Published:** 2021-08-31

**Authors:** Wilson Sim, Wen Hui Lim, Cheng Han Ng, Yip Han Chin, Clyve Yu Leon Yaow, Clare Wei Zhen Cheong, Chin Meng Khoo, Dujeepa D. Samarasekera, M. Kamala Devi, Choon Seng Chong

**Affiliations:** 1 Yong Loo Lin School of Medicine, National University of Singapore, Singapore, Singapore; 2 Division of Endocrinology, Department of Medicine, National University Hospital, Singapore, Singapore; 3 Centre for Medical Education, Yong Loo Lin School of Medicine, National University of Singapore, Singapore, Singapore; 4 Alice Lee Centre for Nursing Studies, Yong Loo Lin School of Medicine, National University of Singapore, Singapore, Singapore; 5 Division of Colorectal Surgery, Department of Surgery, University Surgical Cluster, National University Hospital, Singapore, Singapore; City University of New York Graduate School of Public Health and Health Policy, UNITED STATES

## Abstract

**Objective:**

To understand racial bias in clinical settings from the perspectives of minority patients and healthcare providers to inspire changes in the way healthcare providers interact with their patients.

**Methods:**

Articles on racial bias were searched on Medline, CINAHL, PsycINFO, Web of Science. Full text review and quality appraisal was conducted, before data was synthesized and analytically themed using the Thomas and Harden methodology.

**Results:**

23 articles were included, involving 1,006 participants. From minority patients’ perspectives, two themes were generated: 1) alienation of minorities due to racial supremacism and lack of empathy, resulting in inadequate medical treatment; 2) labelling of minority patients who were stereotyped as belonging to a lower socio-economic class and having negative behaviors. From providers’ perspectives, one theme recurred: the perpetuation of racial fault lines by providers. However, some patients and providers denied racism in the healthcare setting.

**Conclusion:**

Implicit racial bias is pervasive and manifests in patient-provider interactions, exacerbating health disparities in minorities. Beyond targeted anti-racism measures in healthcare settings, wider national measures to reduce housing, education and income inequality may mitigate racism in healthcare and improve minority patient care.

## Introduction

In recent times, massive anti-racism protests around the world, following the unjust death of Mr George Floyd at the hands of a police officer in the United States, have called for greater scrutiny of existing racial injustices across all institutions, including within the public health sphere. Racial health disparities have been long standing, as evidenced by the landmark report from the Institute of Medicine (IOM) in 2003. Minorities were documented to receive fewer procedures and poorer quality medical care than the majority, even after controlling for confounders [1]. The severity of racial health inequities is further reflected in the COVID-19 pandemic where racism in healthcare has been purported to be a significant driving force of the disproportionately high mortality rates in minorities [2]. In a recent article by Devakumar et al, racism was declared to be a public health emergency of global concern [3], supported by literature which presents pronounced evidence on unequal healthcare delivery for minority groups in the US [4, 5].

Although over the years, increased awareness of racism have prompted denouncement of overtly racist actions, multiple studies have reported observations of subtle, aversive racism among physicians [6, 7]. This has been found to impact treatment decisions, corroborated by literature which revealed that even after controlling for confounding variables such as severity of illness, insurance, and income, Black males are less likely to receive medical procedures compared to their White counterparts [8]. Such interpersonal racism has been observed to pervade various healthcare domains. In dentistry, although patients presented with similar symptoms, there was a greater likelihood of Black patients being offered tooth extraction instead of restorative root canal treatment, reflecting unconscious racial bias in treatment planning decisions [9]. Similarly, in cardiology, minority patients were less likely to be referred for cardiac catheterization despite residents being presented with standardized hypothetical patients [10]. In the general hospital setting, Black patients’ pain were also often underestimated and undertreated by residents who held false beliefs that Black patients have higher pain tolerance than other patients [11]. Studies have consistently shown that these negative experiences of racism not only create undue stress for minorities [12], but fuel deep mistrust in the healthcare system, therefore perpetuating a vicious cycle of poor health outcomes [12, 13]. Racism has been associated with poorer medication adherence and underutilization of healthcare services by minority patients [14, 15].

Recognizing that racism unfairly penalizes minorities, policy statements and funding have been increasingly directed towards addressing institutional racism in medical care [16]. Despite these measures, little headway has been made in achieving racial equality. Thus, this has brought attention to racial discrimination at the interpersonal level stemming from healthcare providers’ explicit and implicit racial biases [17]. To date, quantitative studies and systematic reviews have presented well-founded evidence of unconscious bias in physicians against minority races but fail to inform the nature of prejudicial behaviours in the healthcare system. Conversely, qualitative studies which capture the experiences of minority patients can increase awareness of patient-provider racism in the healthcare system to inspire changes in how healthcare providers treat minority patients. Hence, we sought to conduct a systematic review of qualitative studies to shed light on racial bias in the healthcare system in order to attenuate health disparities among minorities and address racism as a healthcare crisis.

## Materials and methods

### Search strategy

This qualitative systematic review was conducted in accordance to the Preferred Reporting Items for Systematic Reviews and Meta-Analyses (PRISMA) statement [18] and ENTREQ [19]. The following electronic databases were systematically searched from inception till 25 June 2020: Medline, CINAHL, PsycINFO, and Web of Science Core Collection. The search strategy is attached in S1 File. Articles deemed potentially relevant underwent a title and abstract sieve, followed by a full text review for inclusion by two independent authors. The final inclusion of the articles was based on consensus between the two authors.

### Study selection and eligibility criteria

Authors individually identified studies that met the following inclusion criteria: 1) qualitative or mixed methods methodology, 2) perceptions of racial bias or racial blindness from patient or provider perspectives; and 3) studies related to racial disparity in the healthcare setting. Only original, peer-reviewed articles written in or translated into the English language were considered. Commentaries, letters to the editor, reviews, conference abstracts, and grey literature were excluded. Two authors independently conducted full text review and any disagreements were discussed till a consensus was reached.

### Data extraction and analysis

The data extraction sheet included origin and year of publication, objective, methodology, demographics (race and ethnicity of majority and minority, gender, age, sample size) of participants and primary findings from the included articles. Coding was carried out verbatim only for quotes from patients and providers to depict the minority experience in healthcare delivery. Thematic synthesis was employed to review the data, using the Thomas and Harden framework which comprises three stages of detailed synthesis: line-by-line coding of the primary text, construction of descriptive themes, and the development of analytical themes [20–24]. According to Thomas et al, the inductive approach allows for the most empirically grounded and theoretically interesting factors arising directly from the raw data rather than a priori expectations or models. The primary text was first extracted and organized into a structured proforma, before inductively derived codes were cross examined with the raw data, given context and original authors’ interpretations. During this process, the original authors’ interpretations were taken into account to minimize bias as researchers may intuitively search for data that confirm his/her personal experience and beliefs. This phase of the analysis was equally iterative, moving back and forth between the codes and the original articles to ensure the robustness of the analytical process. Subsequently, descriptive themes were independently formed based on repeated readings of the mutually agreed codes to identify and group recurrent ideas. The descriptive themes were then further refined until a consensus was reached. Analytical themes were distilled by forming a relational quality among descriptive themes to synthesize fresh perspectives and explanations beyond primary data. Discussions were held between authors for clarification and comparison of primary findings and final synthesis.

### Quality assessment

Quality appraisal of included studies was conducted by using the Critical Appraisal Skills Programme (CASP) [25]. The CASP Checklist consists of 10 items developed to assesses the trustworthiness, relevance and results of published papers. Quality assessment was independently conducted by two authors, with disagreements being resolved by discussion until consensus were reached. The results of quality assessment did not result in exclusion of any studies, but were instead used to add to the collective rigor of the synthesis. The PRISMA 2009 Checklist (S2 File) was used to ensure the completeness of this review.

## Results

**Fig 1:**
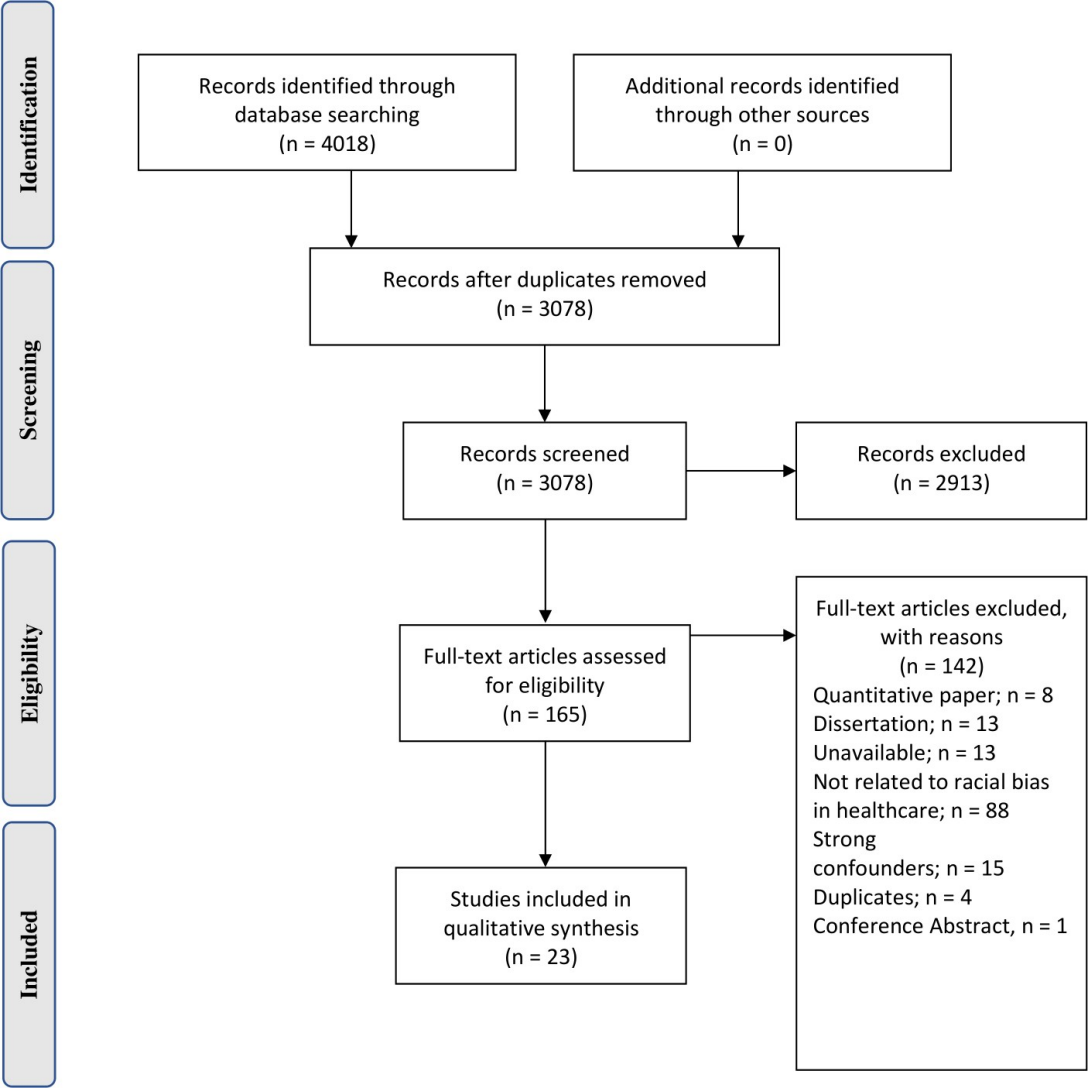
PRISMA flow diagram.

Electronic search results identified a total of 4,018 articles. 3,078 remained after duplicate removal and 165 articles were selected for full text review, of which 23 articles met the inclusion criteria. (Fig 1) In total, there were 1,006 participants which comprised 727 healthcare users and 249 healthcare providers. The age of participants ranged from 24 to 89 years old. The included studies were conducted in 6 different countries: 15 in the United States [8, 26–39], 3 in the United Kingdom [40–42], 2 in Australia [43, 44], 1 in Canada [45], Spain [46] and Hong Kong [47] respectively. 11 studies reported findings from the African American community, 2 each from the Pakistani, Indigenous Australian and South Asian communities and 1 each from the African Caribbean, Latin American, Native American, Roma and Iranian communities. The characteristics of the included papers are presented in S1 Table. The quality of included articles by CASP can be found in S3 File. In the thematic synthesis of codes regarding the perpetuation of racial bias in healthcare delivery, 2 analytical themes were generated from minority patient perspective: *alienation of minority patients*, *labelling of minority patients* while 1 analytical theme was generated from healthcare provider perspective: *perpetuation of racial fault lines by providers*.

### Alienation of minority patients

#### Racial supremacism

*“I was feeling like he was trying to belittle me and my intellect*.*”* -Minority patient of unspecified race in the US [34].

Minority patients reported that they were often stereotyped to be of lower intelligence, elaborating on how providers doubted their ability to understand information [26, 34, 46], and did not provide sufficient information regarding their treatment, leaving them feeling uninformed [26, 28, 46]. Minority patients felt that their intellect was belittled [34], when providers spoke to them in an overly simplified manner or forced views upon them [26, 46]. Additionally, they echoed the sentiment that healthcare providers were condescending towards them. This perception arose due to the raised voices, curt tone and dismissive mannerisms when healthcare providers attended to the minority patients [26, 29, 31, 34, 40, 45]. In areas where the White community is the majority, minority patients further observed that healthcare providers were significantly more polite to White patients [31, 34, 45], but more disrespectful towards them [31, 32, 34, 37, 39, 40, 46]. Minority patients were also subjected to overt racism from providers in the forms of rude facial expressions [40], reluctance to make skin contact during medical examinations [28, 32, 41], avoidance of eye contact and cold body language [26, 34]. This resulted in feelings of being belittled, hated or embarrassed [26, 34, 40, 45].

#### Less empathetic care received

*"They feel that (nurses) do not want to bother with them*. *They are not wanted*. *They feel that nurses are not liking them*. *Sometimes*, *what nurses do is not obvious but it is underhand*. *Those (patients) who cannot speak English get into trouble*, *and they get a bit bullied as well*.*"*—Pakistani patient in the UK [40].

Minority patients reported that healthcare providers ignored or rushed them during their clinical interactions [32, 40, 41, 44, 47]. Some patients further stated that they received less priority and were unfairly skipped over by patients of perceived privileged races [29, 31, 32, 34, 38]. Likewise, other minority patients felt that they were treated more harshly, with accounts of rough physical treatment from healthcare providers [34]. While healthcare providers engaged patients from the majority community with cheerful and sociable conversations, their dispositions became more formal or hostile when interacting with minority patients [31, 32, 34, 37, 46].

### Labelling of minority patients

#### Assumptions of class

*“I’m suppose to look like I got some money*, *cause if a Black person come in there dirty or looking ragged then that is the kind of treatment*. *You looking poor*, *and raggedy then you gonna get some raggedy treatment*.*”*—Black patient in the US [26].

Most minority patients recalled being stereotyped as having low socioeconomic status [26, 28, 34, 46], less educated [46], having poor living conditions [26], or needing financial support [34]. They also recounted being judged more harshly for their appearance and felt compelled to dress well for better treatment [26]. They felt that providers assumed they were unable to afford medical services [28, 46], and consequently, gave half-hearted medical treatments [26, 34], such as not offering the full range of treatment options [8, 38]. On the other hand, there was a small number of patients who did not perceive racial discrimination in their clinical encounters. They felt that providers focused on treating their illnesses, without taking into account their skin colour [30, 45]. Most of these patients were Iranian immigrants of higher socioeconomic status (SES) [30], suggesting the role of SES in affecting perceptions of racism.

#### Assumptions of negative behaviours

*“But* …*he was a huge*, *darker skinned Black male*, *and I think that people saw him as intimidating*. *And it was just easier to just kind of bypass him and do the minimal that you had to do*.*”*—Black registered nurse in the US [8].

Minority patients reported that providers perceived them to be difficult to appease, dangerous and were afraid of patients [42, 46], which in turn, made patients uncomfortable [26]. Some African American patients were also denied medications due to race-based assumptions by healthcare providers that they were drug users [32, 37]. In the same vein, patients concurred and felt that providers treated them like a homogenous group without varying needs or beliefs [44–46]. For instance, providers made sweeping generalizations that African American patients had overactive sex lives [26, 29].

### Perpetuation of racial fault lines by providers

#### Differential treatment of minority patients

*"I’m leaning towards more than the physician is empathizing more for the White patient because he has more of a connection with him*‥ ‥ *Most doctors who are very good doctors*, *and otherwise nice people*, *are simply doing less for the Black patient because they have this unconscious racism*.*”*—Black primary care/internal medicine doctor in the US [27].

Healthcare providers professed that they were less empathetic towards minority patients [27, 33, 35], even losing their temper more easily [47], because they were less able to connect to patients of a different race [27]. This is further exacerbated by the time constraint these providers face [33, 35], pressuring them to limit their interactions with minority patients unintentionally [27]. Furthermore, some healthcare providers concurred that they viewed minorities as more intimidating due to their appearance which limited their effort to engage with them or offer therapy [8, 42]. They acknowledged their failure to understand the differing needs of minority patients to administer individualized care [8, 35, 36, 40]. Instances of overt racism were also reported by providers who observed that racial minorities were sometimes referred to using racially derogatory labels [47]. These discriminatory healthcare encounters can perpetuate racial fault lines which are currently unobvious problems that can eventually result in further tension and conflicts between the minority and majority population. These fault lines may have arose from fundamental differences in opinions, or underlying divisive issues tracing back to the historical origins of White supremacy.

#### Shifting the blame onto minority patients

*“To be honest*, *some patients have a chip on their shoulder about colour and a lot of fuss is made up over nothing* …*I am sorry to say*.*"*—White registered nurse caring for Pakistani patients in the UK [41].

Providers frequently labelled minority patients as less compliant with treatment and felt that they lacked ownership over their health [27, 35, 43, 45]. These providers tended to blame health disparities [27, 36, 45], or unsuccessful treatments [41], on minority patients’ poor behaviours, instead of unequal treatment arising from racism [35]. Some providers stated that they treated all patients equally [33, 36], while others perceived minority patients to be oversensitive, holding the view that these patients misinterpret innocent health encounters as racist due to their past experiences of racism [26, 43]. Providers who denied racism also reported that minorities played the race card unnecessarily [35, 41], which is the act of trying to gain sympathy or special treatment because of their race [48], in some instances being oversensitive or victimizing themselves [35, 41]. Although research has hypothesized that minorities may respond with disproportionate negativity to innocuous events, there is in fact conclusive evidence that reveals how minorities experience microaggressions more frequently [49]. Thus, such perceptions by providers may lead to a lack of responsibility and inaction in addressing personal biases.

## Discussion

To our knowledge, this is the first study to systematically review the perspectives on racism in healthcare from ethnic minorities across different countries. Racial bias manifests in healthcare delivery through the alienation of minority patients, labelling of minority patients and perpetuation of racial fault lines by healthcare providers. Importantly, ethnic minority groups are heterogeneous populations in terms of their ethnicity, socio-demographic status, acculturation level, and belief systems. While the findings should be taken with caution across different settings, the collective examination of these individual experiences synthesizes commonalities from disparate evidence, and clearly illuminates the different aspects of racial discrimination in healthcare. With the elimination of racism at the forefront of the global agenda, urgent assessment of existing measures is greatly warranted for healthcare providers to extend fair and equal access to quality care for all patients so that vulnerable communities do not continue to fall through the gaps. For the convenience of this review, the term “White” was used to collectively describe people of pale skin, instead of “Caucasian” which has been found to be an outmoded misnomer in racial nomenclature with little value in racial discussions.

In the included studies, both accounts of healthcare providers and minority patients corroborated to show that minority patients were often subjected to labelling where assumptions were made about their class, behaviours and needs [8, 26, 28, 29, 32, 34–37, 42, 44–46]. This may be explained by research which demonstrates how under limited time and imperfect information, confirmation bias occurs as providers fall back on innate beliefs associated with patients’ social categories [50]. Providers should strive to avoid labelling of patients as it has been reported that they often overapply such population statistics to individual patients [51], even if the stereotypes were grounded on epidemiology. Additionally, minority patients perceived less empathy from healthcare providers [31, 32, 34, 37, 46], which corresponded to providers’ perspective on the differential treatment of minority patients [8, 27, 33, 35, 36, 40, 42, 47]. These two themes have a causal relation where providers were less able to put themselves in the shoes of minority patients to vicariously experience their circumstances, resulting in them being less able to connect to minority patients and express empathy towards them. In fact, barriers to empathy is highly prevalent in clinical settings; general practitioners claim that protocol-driven care impede genuineness in communication and time pressure hinders communication [52]. For instance, the heavier workload in emergency departments can cause high tension situations which reduce empathetic abilities of healthcare providers [53]. Providers should endeavour to communicate empathy and interact more deeply with minority patients which has been shown to benefit patient health and is part of an evidence-based practice [54].

The pigeonholing of minority patients may contribute to inadequate medical treatment like exclusion from certain therapies [8, 32, 38, 42, 45, 46], or poor communication and service from healthcare providers [8, 35, 36, 46]. This is supported by past systematic reviews which found that providers’ innate prejudices often result in lower quality of care for racial minorities [4, 55, 56]. Furthermore, perceived racism has been found to affect mental and physical health negatively [57, 58], while breeding mistrust in minority patients who may respond by not complying to treatment plans [14, 15]. This may eventually translate into worse health outcomes for minority patients [28, 34, 42], reinforcing providers’ perceptions of them as being less responsible for their health [27, 35, 43, 45], which perpetuates a vicious cycle. Providers should actively perform self-reflection as objectively as possible beyond implicit bias assessments such as the Implicit Association Test [59]. While there is only preliminary evidence on the efficacy of self-reflection to reduce implicit bias [60], the action of observing and analysing oneself can promote increased cognition of biased behaviours and is an important first step.

It is vital for healthcare providers to acknowledge that racism in healthcare continues to be a pertinent problem and actively reflect on how their actions may affect the emotions and care of minority patients. By committing the effort to understand the issue and address innate prejudices against other races, healthcare providers can avoid the tendency to label minority patients under imperfect information or time constraint. Providers should also be more mindful of their physical and verbal communication and avoid unequal treatment between minority and majority patient group in terms of politeness, patience and willingness to engage in conversations. An increased sensitivity towards innocent situations where misunderstandings could arise, such as minority patients getting skipped over, would ultimately aid in creating an inclusive healthcare environment.

With a shift towards inclusivity, several countries have implemented frameworks to achieve greater racial equality in healthcare (S2 Table). These interventions originate mainly from developed countries where there is significantly more literature documenting racial disparities in healthcare, thus, increasing national awareness and priority in tackling these issues [61–66]. Most countries do not directly address racism towards patients but propose guidelines on reducing health disparities among minorities [62–64, 66]. One of the common aims is to increase racial and ethnic diversity in their healthcare workforce [62–64, 66]. Cross-cultural exchanges among providers may address unfounded racial assumptions and dispel fear of minorities [8, 26, 42, 46]. Some countries also seek to address inadequate medical treatment [8, 26, 34, 38], using evidenced-based guidelines for management of chronic diseases in racial minorities [64, 66], and increasing minority health research [61, 66]. However, these measures may not translate into individualized care for minorities as stereotypes of class and behaviors remain unaddressed [8, 28, 32, 35–37, 46]. Several papers detail further plans to deliver timely, patient-centered care to minorities and better communication [61, 64, 66]. Analysis of these plans was limited due to the lack of quantifiable and definitive steps to achieve these goals. It is important to note that without conscious efforts to address the innate tendency to alienate, biased attitudes may continue to translate into clinical encounters with minorities through subtle body language [26, 34], or hostile demeanour [31, 32, 34, 37, 46]. Additionally, only two programs outline goals of educating providers on racial discrimination [61, 65], while most interventions focus solely on training providers’ cultural competency [62, 64–66]. Although cultural incompetency is interlinked with perceived racism, it is important to note that they are ultimately different issues and should not be conflated [67]. Cultural competency training alone may not fully address providers’ unawareness of implicit biases, allowing racial color-blindness and tendency to blame minorities for health disparities to persist [8, 26–29, 32–37, 42–46]

Overall, the measures to tackle racism in healthcare is a work in progress across all nations. There is an urgent need for more concrete and actionable anti-racism programs like incorporating bias curriculum early into medical education and training [68], to signal the strong commitment of institutions to tackle racism and close racial disparities in the healthcare setting, spearheading a paradigm shift. Additionally, approaches should be aimed at promoting interracial understanding through dialogue sessions or feedback channels for minority patients [69, 70], where difficult conversations on a sensitive topic can take place safely. Improving interracial understanding would eliminate the various assumptions of minority patients that result in poorer medical treatment and service. Availability of feedback channels for minority patients may also increase accountability for providers to act in a non-discriminatory and professional manner, promote further dialogues about racism and decrease racial blindness.

The included studies in this paper originated mainly from Western countries [8, 26–46], but there was a paucity of literature from the Asian perspective (S1 Table). Considering how racism is similarly pervasive in many Asian countries, the lack of literature may be attributed to underreporting as open discourse about highly sensitive topics like racism may be limited by censorship which is more common among Asian countries [71, 72]. Therefore, further qualitative research needs to be conducted to yield insights into the nature of racism that minority patients face in Asian healthcare systems. By looking introspectively at Singapore, a multiracial Asian society, as a case study, self-reflection into areas of success and improvement for tackling racism may yield critical learning points to devise concrete ways forward in achieving racial equality in healthcare. In 1969, after a series of deadly racial riots broke out between Malays and Chinese in Singapore, governmental efforts to build a harmonious multiracial society were increased. Measures implemented included racial integration through conscription, racial quotas for housing estates, education to promote meritocracy by offering equal opportunities for all races [73], and ensuring that minority races are represented in the government through the Group Representation Constituencies (GRCs) [74]. Beyond anti-racism measures, governmental policies addressed larger racial disparities in the form of education and housing, factors which can exacerbate discrimination or feelings of discrimination [75]. The collective awareness of the fragility of racial harmony served as a significant driver in making multiculturalism a core tenet of the nation’s social fabric. This multi-pronged approach by the government translated into heightened race-consciousness among citizens which has allowed Singapore to see marked improvements in race relations over the years with better racial integration and less racial inequities [76, 77]. The key to addressing racism in healthcare may extend beyond healthcare boundaries as governmental efforts to promote multi-culturalism and address wider racial disparities in the form of housing, education and income inequality could have a crucial impact on eliminating racism in all spheres, since class and race prejudice are highly intertwined.

### Limitations

Limitations should be taken into consideration when interpreting these results. Firstly, only articles written in or translated into the English language were included. However, racial minorities across Europe and Asia may not speak English, so relevant studies may have been published in local languages only. Thus, the magnitude of racism in healthcare may be underestimated. Compared to African American minorities whose perspectives were well captured due to the large number of studies from the United States, there were insufficient studies on minorities receiving medical care in Asian countries whose experiences may differ and cannot be accurately represented by the findings. Considering the paucity of data, more studies including but not limited to various ethnic minorities in Asia and Aboriginal Australians should be conducted in the future to better examine the experiences of these minority groups. Additionally, the differences in extent and forms of racism between developed and developing countries are not explored here due to a lack of literature. Due to the lack of granularity of data in the included studies on the SES of participants, further analysis, for instance, subgrouping could not be conducted to better understand how SES may lead to perceptions of racism. With race and ethnicity shown to be highly intertwined with SES, prejudicial behaviours of healthcare providers could arise from class discrimination, as corroborated by existing literature which illustrates how patients with lower SES perceived decreased quality of care [78]. Thus, SES is a confounder for the findings of this review. A largely inductive approach was undertaken to analyse the extracted quotes with their contexts embedded. Unlike deductive analysis, a pre-existing coding frame driven by researchers’ theoretical interest was not utilized. It is important to note that researchers cannot free themselves of their theoretical and epistemological commitments, and data are not coded in an epistemological vacuum. However, themes derived from inductive analysis is driven by raw data, possibly providing a richer description of minority’s experiences [79]. Lastly, the distinction between explicit and implicit bias, though explored in the results, is often difficult to make due to the qualitative nature of the primary data. Importantly, in a recent study by Daumeyer et al [80], he demonstrated that labels of implicit bias in racial discussions may paradoxically reduce individual culpability for discriminatory behaviour due to models of behavioural attribution where perpetrator of unintentional racism is viewed as less morally responsible. Therefore, this study which focuses on the perspectives and experiences of minority patients, encourages providers to examine all biases to take accountability for racially discriminatory behaviours.

## Conclusion

This systematic review has analyzed the experiences of minority patients and has yielded fresh insights on the forms of racial bias that minority patients continue to face today. Though policy makers and healthcare institutions have attempted to reduce health disparities, racism continues to persist at the interpersonal level in an insidious and implicit form. To protect the fundamental right to quality healthcare for all, healthcare institutions need to urgently establish targeted anti-racism programs. As we head into a new decade, this review serves as a call-to-action for institutions and providers to reflect deeply on racial injustices that continue to plague global healthcare systems and to actively work towards offering non-discriminatory and sensitive care, in the journey towards achieving racial equality and dignity for all.

## Supporting information

S1 Table Characteristics of included papers. (DOCX)

S2 Table Overview of programs for racial equality in healthcare across countries. (DOCX)

S1 File Medline search. (DOCX)

S2 File PRISMA 2009 checklist. (DOC)

S3 File CASP qualitative checklist. (DOCX)
