# Supplementary material for: The perspectives of health professionals and patients on racism in healthcare: A qualitative systematic review
Source: PLoS One. 2021 Aug 31;16(8):e0255936. doi: 10.1371/journal.pone.0255936 (PMC8407537; doi:10.1371/journal.pone.0255936)
Supplement: S1 Table — (DOCX) [file pone.0255936.s001.docx]

**S1 Table. Characteristics of Included Papers**.

| **Author** | **Year** | **Country** | **Participants** | | | | | **Methodology** | **Perspective** | **Objective** |
| --- | --- | --- | --- | --- | --- | --- | --- | --- | --- | --- |
|  | | | **Number** | **Race/ethnicity of Majority** | **Race/ethnicity of Minority** | **Gender (Female %)** | **Age**  **(Mean/ Range)** |  |  |  |
| Cortis *et al.* | 2000 | UK | 55 | White | Pakistani Muslim | 45.5 | 44-66 | Qualitative; FGDs and Individual Interviews | Patient | To explore the perceptions and experiences with nursing care: A study of Pakistani (Urdu) communities in the United Kingdom |
| Mclean *et al.* | 2003 | UK | 30 | White | African-Caribbean | NA | NA | Qualitative; FGDs and Individual Interviews | Both | To detail the local perspectives on interactions between the community and health services, through which inequalities are experienced |
| Cortis *et al.* | 2004 | UK | 30 | White | Pakistani | 86.7 | NA | Qualitative; Semi-Structured Interviews | Provider | This paper reports an investigation into the experiences of Registered Nurses caring for hospitalized Pakistani patients in the United Kingdom |
| Johnson *et al.* | 2004 | Canada | 122 | White | South-Asian | 92.6 | 47.6, 20-80 | Qualitative; FGD and Open-Ended Interviews | Both | To explore the interactions between health care providers and South Asian immigrant women to describe othering practices and their effects |
| Benkert *et al.* | 2005 | US | 20 | White | African-American | 100 | 53.5 | Qualitative; Semi-Structured Interviews | Patient | To describe African American women’s perceptions of prejudice in health care and the strategies used to cope with those experiences |
| Hatzfeld *et al.* | 2008 | US | 16 | White | African-American | NA | NA | Qualitative; FGDs | Patient | To describe African-American's perceptions of health disparities |
| Clark-Hitt *et al.* | 2010 | US | 26 | White | African-American | 53.8 | NA | Qualitative; Semi-Structured Interviews | Provider | To gain better insight into how race may affect treatment |
| Greer *et al.* | 2010 | US | 37 | White | African-American | 86.5 | 52.11 | Qualitative; FGDs | Patient | To examine African American patient perceptions of racial discrimination in clinical encounters |
| Sims *et al*. | 2010 | US | 50 | White | African-American | 100 | 40-70+ | Qualitative; Group Interviews, Individual Interviews | Patient | To report the first-hand perspectives of older Black women within healthcare encounters that impact the trajectories of health-seeking behaviors |
| Durey *et al.* | 2012 | Australia | 3 | White Anglo-Australian | Indigenous Australians | 66.7 | NA | Qualitative; Interviews | Provider | To explore the effects of ‘White’, Anglo-Australian cultural dominance in health service delivery to Indigenous Australians |
| Martin *et al.* | 2012 | US | 15 | White | Iranian | 53.3 | 73.9 | Qualitative; In-Depth Interviews | Patient | To explore older Iranian immigrants’ perceptions/experiences of discrimination in their encounter with the American health care system |
| Tajeu *et al.* | 2015 | US | 92 | White | African-American | 51.1 | 48.5 | Qualitative; FGDs | Patient | To qualitatively assessed patients’ perceptions of discrimination and patient satisfaction in the health care setting specific to interactions with nonphysician health care staff. |
| Cuevas *et al.* | 2016 | US | 60 | White | African-American | 41.7 | 56.8 | Qualitative; FGDs | Patient | To explore participants’ experiences and relationships in health care settings |
| Worrall-Carter *et al.* | 2016 | Australia | 10 | White Anglo-Australian | Indigenous Australian | 40 | NA | Qualitative; In-Depth Interviews | Patient | To explore Aboriginal patients’ lived experiences of cardiac care at a major metropolitan hospital in Melbourne |
| Cuevas *et al.* | 2017 | US | 142 | White | African-American | NA | NA | Qualitative; FGDs | Patient | To gain a better understanding as to whether disparities in patient–provider relationships arise from ethnic minority patients being treated differently than European American patients while they would prefer to be treated the same, or whether disparities arise when ethnic minority patients are treated the same as European American patients while they would prefer to be treated differently |
| Plasime *et al.* | 2017 | US | 11 | White | African-American | 45.5 | NA | Qualitative; Semi-Structured Interviews | Provider | To explore health providers’ formative personal and professional experiences with race and Black men as a way to assess their potential influence on interactions with Black male patients |
| Aiello *et al.* | 2018 | Spain | 17 | Spanish | Roma | NA | 34.5 | Qualitative; Semi-Structured Interviews | Patient | To explore the barriers that the Roma face in accessing the healthcare system, reflecting on how these barriers are accentuated by the existing anti-Roma prejudices and institutional arrangements that do not account for minority cultures |
| Cunningham *et al.* | 2018 | US | 46 | White | Of Colour | NA | NA | Qualitative; FGDs and Semi-Structured Interviews | Provider | To understand: 1) how colorblindness “shows up” when health care professionals aim to promote equity; 2) how their colorblindness informs (and is informed by) clinical practice; and 3) ways to overcome colorblindness through strategies grounded in CRT |
| Gollust *et al.* | 2018 | US | 53 | White | Black, Latino | 50.9 | NA | Mixed Method; Semi-Structured Interviews | Provider | To understand providers’ perceptions of causes of racial health care disparities |
| Gonzalez *et al.* | 2018 | US | 74 | White | Black, Latino | 59.5 | 41.8 | Qualitative; FGDs | Patient | To explore patients’ perceptions of bias, and suggestions for restoring relationships if bias is perceived |
| Purtzer *et al.* | 2019 | US | 11 | White | Native American | NA | 39-67 | Qualitative; Semi-Structured Interviews | Provider | To examine health disparities within the context of patient/nurse relationships |
| Connell *et al.* | 2019 | US | 64 | White | African-American | 62.5 | 45-64 | Qualitative; FGDs | Both | Compares and contrasts perspectives of lay community members, volunteer community health advisors (CHA), and health care providers related to structural and interpersonal barriers to health care seeking and provision among African American adults experiencing health disparities in the rural Mississippi Delta. |
| Vandan *et al.* | 2020 | Hong Kong | 22 | Hong Kong Chinese | South-Asian | 40.9 | 34, 24-78 | Qualitative; Interviews | Provider | This study explores the perceptions of and challenges faced by Hong Kong healthcare professionals in the provision of culturally appropriate care to South Asian ethnic minority patients. |

Legend:

NA – Not Applicable

FGD – Focus Group Discussion
