# Supplementary material for: The perspectives of health professionals and patients on racism in healthcare: A qualitative systematic review
Source: PLoS One. 2021 Aug 31;16(8):e0255936. doi: 10.1371/journal.pone.0255936 (PMC8407537; doi:10.1371/journal.pone.0255936)
Supplement: S2 Table — (DOCX) [file pone.0255936.s002.docx]

**S2 Table: Overview of Programs for Racial Equality in Healthcare Across Countries.**

| **Country** | **Name of Program** | **Organisation** | **Aims** | **Objectives and Overview** |
| --- | --- | --- | --- | --- |
| United States of America | Department of Health and Human Services Action Plan to Reduce Racial and Ethnic Health Disparities | Department of Health and Human Services | To build a nation free of disparities in health and health care. | - Reduce disparities in health insurance coverage and access to care. - Reduce disparities in the quality of health care by removing barriers to the timeliness, patient-centeredness of care, and the equitable use of evidence-based clinical guidelines. - Increase the ability of all health professions and the healthcare system to identify and address racial and ethnic health disparities. - Promote the use of community health workers and promotoras. - Increase the diversity of the public health workforces. - Reduce disparities in population health by increasing the availability and effectiveness of community-based programs like health education and preventive services. - Conduct and support research to inform disparities reduction initiatives. |
| United Kingdom | The Equality Delivery System (EDS2) | National Health Service – Equality and Diversity Council | To create better health outcomes, improved patient access and experience, a representative and supported workforce and inclusive leadership | - Access and meet individual health needs in appropriate ways. - Extend screening, vaccination and health promotion initiatives to reach all communities. - Promote access to primary cares services for all. - Ensure patients are informed and supported in their decisions about their care. - Create positive experiences for patients. - Handle complaints about services efficiently and respectfully. - Create a more representative workforce. |
| Spain | Comprehensive Strategy against Racism, Racial Discrimination, Xenophobia and related Intolerance | Ministry of Labour and Immigration | 1. Promote access to health care without any kind of discrimination. 2. Establish effective mechanisms to monitor and eliminate racially or ethnically motivated discrimination in the health care system. 3. Enable cultural diversity training for healthcare professionals. 4. Coordinate prevention and protection programmes and action against racism and xenophobia to be carried out among professionals, care staff, researchers and private companies. | - Develop plans and programmes by the Public Health Administrations, according to their competences, in order to adapt the health system to incorporate the axis of non-discrimination on racially and ethnically motivated grounds. - Develop intercultural health mediation measures and peer education in the healthcare sphere. - Promote cultural adaptation of teaching and information and awareness-raising materials, and preparation of training material. - Encourage capacity-building and training in racially and ethnically motivated discrimination of professionals working in health care professions. - Facilitate translating of documents and interpreting - Hold dialogues with professionals, health care staff, researchers and private companies to develop measures to prevent racially or ethnically motivated discrimination in the health care system. - Create mechanisms for denouncing, protecting and assisting victims of racist or xenophobic incidents. |
| Australia | AH16 Aboriginal and Torres Strait Islander health | The Royal Australian College of General Practitioners | To enable General Practitioners to work respectfully, in collaboration with and provide quality care to Aboriginal and Torres Strait Islander peoples and when required, to advocate for individuals and their communities. | - Improve communication and doctor-patient relationship by working with cultural mentors and utilizing language services. - Develop effective therapeutic relationships by identifying negative stereotypes and common cultural influences on health behaviours. - Devise strategies to empower patients to achieve better health outcomes. - Identify and address diagnostic uncertainty related to cultural beliefs. - Use evidence-based preventive and population health approaches to reduce health inequalities. - Support health research and education. |
| Israel | Racism in Healthcare | Ministry of Health - Committee on the Elimination of Racial Discrimination and Exclusion | To realize the healthcare system's ability to be the leader in charge of the struggle to eradicate racism, discrimination and exclusion in the Israeli society. | - Formulate a joint social convention through dialogue circles with diverse social groups, with patients, with caregivers, with worker unions. - Appoint a designated official whose role will include handling individual complaints alongside locating institutionalized occurrences and handling them. - Train and educate the in-field students and teams. - Make the option for filing a complaint present, accessible and simple. - Leadership and management: guide, incentivize and encourage senior officials on the matter. - Host a public awareness campaign to create trust and an open atmosphere that is anti-racist and that promotes an egalitarian reality. - Redesign "volatile spaces" such as emergency room departments or maternity wards by physical and organizational means (such as family visits procedures). - Establish and promote research on racism. - Increase efforts to improve the diversity in employment. |
| Ireland | National Traveller and Roma Inclusion Strategy 2017 – 2021 | Department of Justice and Equality | 1. Travellers and Roma have improved access, opportunities , participation rates and outcomes in health care system 2. Reduce health inequalities 3. Deliver culturally appropriate care 4. Reduce rate of suicide and mental health problems among Travellers and Roma | - Ensure that there is Traveller and Roma representation on their national and local health-related structures relating to Travellers and Roma, as appropriate. - Address the prevalence, range and treatment of chronic health conditions amongst travellers e.g. diabetes, asthma, cardiovascular and circulatory conditions, poor mental health and suicidal ideation. - Promote immunisation uptake among members of the Roma community, and support Roma women to access maternal health services in a timely and appropriate manner. - Explore the inclusion of certain training elements as appropriate on Traveller health status and Traveller and Roma cultural awareness on the undergraduate and graduate curricula for health professionals. - Design and disseminate culturally appropriate, accessible information and healthcare materials in partnership with Traveller and Roma organisations. - Assess the primary care and basic needs of vulnerable Roma. - Support and train healthcare staff to use clear language in dealing with diagnosis and treatment options. |
