# Supplementary material for: The perspectives of health professionals and patients on racism in healthcare: A qualitative systematic review
Source: PLoS One. 2021 Aug 31;16(8):e0255936. doi: 10.1371/journal.pone.0255936 (PMC8407537; doi:10.1371/journal.pone.0255936)
Supplement: S1 File — (DOCX) [file pone.0255936.s003.docx]

**S1 File. Medline Search.**

1. exp Racism/
2. (rac* or unconscious bias or implicit bias or explicit bias or ((discriminat* or bias* or prejudic* or stereotyp* or microaggression* or disparit*) adj5 (of colour or minorit* or black* or africanamerican* or african american* or african* or jew* or asian* or hispanic* or latino* or latin american* or roma*))).tw.
3. 1 or 2
4. exp Health Personnel/ or exp Attitude of Health Personnel/
5. (((healthcare or health care or care) adj (provider* or personnel*)) or nurse* or doctor* or careprovider* or physician*).tw.
6. 4 or 5
7. exp Qualitative Research/
8. ((("semi-structured" or semistructured or unstructured or informal or "in-depth" or indepth or "face-to-face" or structured or guide) adj3 (interview* or discussion* or questionnaire*)) or (focus group* or qualitative or ethnograph* or fieldwork or "field work" or "key informant")).ti,ab. or interviews as topic/ or focus groups/ or narration/
9. 11 or 12
10. 3 and 6 and 9
11. Limit 10 to English
