# Supplementary material for: The perspectives of health professionals and patients on racism in healthcare: A qualitative systematic review
Source: PLoS One. 2021 Aug 31;16(8):e0255936. doi: 10.1371/journal.pone.0255936 (PMC8407537; doi:10.1371/journal.pone.0255936)
Supplement: S3 File — (DOCX) [file pone.0255936.s005.docx]

**S3 File. CASP Qualitative Checklist.**

| **Paper** | **1. Was there a clear statement of the aims of the research?** | **2. Is a qualitative methodology appropriate?** | **3. Was the research design appropriate to address the aims of the research?** | **4. Was the recruitment strategy appropriate to the aims of the research?** | **5. Was the data collected in a way that addressed the research issue?** | **6. Has the relationship between researcher and participants been adequately considered?** | **7. Have ethical issues been taken into consideration?** | **8. Was the data analysis sufficiently rigorous?** | **9. Is there a clear statement of findings?** | **Overall assessment*** |
| --- | --- | --- | --- | --- | --- | --- | --- | --- | --- | --- |
| Cortis et al 2000 | Yes | Yes | Yes | Yes | Yes | No | Yes | Yes | Yes | Strong |
| McLean et al 2003 | Yes | Yes | Yes | Yes | Yes | No | No | Yes | Yes | Strong |
| Cortis et al 2004 | Yes | Yes | Yes | Yes | Yes | No | Yes | Yes | Yes | Strong |
| Johnson et al 2004 | Yes | Yes | Yes | Yes | Yes | Yes | Yes | Yes | Yes | Strong |
| Benkert et al 2005 | Yes | Yes | Yes | Yes | Yes | Yes | Yes | Yes | Yes | Strong |
| Hatzfeld et al 2008 | Yes | Yes | Yes | Yes | Yes | No | Yes | Yes | Yes | Strong |
| Clark-Hitt et al 2010 | Yes | Yes | Yes | Yes | Yes | Yes | Yes | Yes | Yes | Strong |
| Greer et al 2010 | Yes | Yes | Yes | Yes | Yes | Yes | Yes | Yes | Yes | Strong |
| Sims et al 2010 | Yes | Yes | Yes | Yes | Yes | No | Yes | Yes | Yes | Strong |
| Durey et al 2012 | Yes | Yes | Yes | No | Can't tell | Yes | Yes | Yes | Yes | Moderate |
| Martin et al 2012 | Yes | Yes | Yes | Yes | Yes | Yes | Yes | Yes | Yes | Strong |
| Tajeu et al 2015 | Yes | Yes | Yes | Yes | Yes | No | Yes | Yes | Yes | Strong |
| Cuevas et al 2016 | Yes | Yes | Yes | Yes | Can't tell | No | Yes | Yes | Can't tell | Moderate |
| Worrall-Carter et al 2016 | Yes | Yes | Yes | Yes | Yes | No | Yes | Yes | Yes | Strong |
| Cuevas et al 2017 | Yes | Yes | Yes | Yes | Yes | Yes | Yes | Yes | Yes | Strong |
| Plaisime et al 2017 | Yes | Yes | Yes | Yes | Yes | No | Yes | Yes | Yes | Strong |
| Aiello et al 2018 | Yes | Yes | Yes | Yes | Yes | No | Yes | Yes | Yes | Strong |
| Cunningham et al 2018 | Yes | Yes | Yes | Yes | Yes | Yes | Yes | Yes | Yes | Strong |
| Gollust et al 2018 | Yes | Yes | Yes | Yes | Yes | No | No | Yes | Yes | Strong |
| Gonzalez et al 2018 | Yes | Yes | Yes | Yes | Yes | No | No | Yes | Yes | Strong |
| Purtzer et al 2019 | Yes | Yes | Yes | Yes | Yes | No | No | Yes | Yes | Strong |
| Connell et al 2019 | Yes | Yes | Yes | Yes | Yes | Yes | Yes | Yes | Yes | Strong |
| Vandan et al 2020 | Yes | Yes | Yes | Yes | Yes | No | Yes | Yes | Yes | Strong |

*The overall assessment was rated on a scale: ‘Strong’, ‘Moderate’, ‘Poor’.
